# Microneedle-Based Analysis Reveals Polarity-Dependent Spatial Regulation of Macrophage Phagocytosis

**DOI:** 10.3390/mi17040413

**Published:** 2026-03-28

**Authors:** Dan Horonushi, Haruka Yuki, Kaho Noumi, Shinya Kato, Kenji Yasuda

**Affiliations:** 1 Department of Pure and Applied Physics, Graduate School of Advanced Science and Engineering, Waseda University, 3-4-1 Okubo, Shinjuku, Tokyo 169-8555, Japan; dan-horonushi@fuji.waseda.jp (D.H.); y.haru.13821@akane.waseda.jp (H.Y.); sean0816@fuji.waseda.jp (S.K.); 2 Department of Physics, School of Advanced Science and Engineering, Waseda University, 3-4-1 Okubo, Shinjuku, Tokyo 169-8555, Japan; kaho.noumi@ruri.waseda.jp

**Keywords:** macrophage, phagocytosis, cell migration, whole-cell polarity, spatial gating, engulfment prioritization, microneedle manipulation, membrane extension, backtracking, sequential phagocytosis

## Abstract

Phagocytosis and migration in macrophages share key regulators, including Rho family GTPases; however, whether phagocytic membrane extension generates a transient, whole-cell polarity that coordinates migration and spatial prioritization of engulfment remains unclear. Here, we investigated the spatiotemporal coupling between membrane extension and cell migration using opsonized microneedles, which enable controlled stimulation together with long-range membrane extension and backtracking dynamics. During single-needle stimulation, membrane extension was tightly coupled to directional migration, whereas membrane retraction showed weaker coupling. In sequential stimulation with two microneedles, ongoing phagocytosis suppressed competing membrane extension at spatially opposite locations, and a reversal in migration direction was accompanied by initiation of membrane extension toward the second needle. Third-needle experiments further revealed a polarized spatial distribution of phagocytic responsiveness across the cell surface. Consistently, uniform stimulation with multiple opsonized microbeads demonstrated sequential, one-at-a-time engulfment even under near-simultaneous target attachment. These results support a model in which phagocytic membrane extension establishes transient, whole-cell polarity that spatially gates engulfment and coordinates whole-cell migration. The microneedle manipulation platform provides a powerful approach for dissecting the spatiotemporal regulation of phagocytosis and for understanding macrophages as adaptive living micromachines integrating mechanical inputs, transient polarity formation, and sequential target processing.

## 1. Introduction

Macrophages are specialized leukocytes that function as first responders in the innate immune system, recognizing and engulfing foreign objects such as microorganisms and particulate matter through phagocytosis [1,2,3,4,5]. Following antigen internalization, macrophages activate downstream immune responses through antigen presentation and cytokine secretion, thereby linking innate and adaptive immunity [6,7]. Because dysregulation of phagocytosis contributes to autoinflammatory diseases, infection susceptibility, and immune evasion by cancer cells, elucidating the mechanisms governing phagocytic target recognition and internalization is of fundamental importance [8,9].

Phagocytosis is generally described by the zipper mechanism, in which ligand–receptor interactions between opsonized targets and cell-surface receptors, such as Fc receptors and complement receptors, drive progressive membrane extension and engulfment [10,11,12,13,14]. This process consists of sequential stages, including target recognition, membrane protrusion, phagocytic cup formation, and internalization, which are regulated by coordinated actin cytoskeletal remodeling and signaling cascades [8,15,16]. Actin polymerization generates protrusive forces that extend the membrane around targets, while actomyosin contractility contributes to engulfment completion and membrane retraction [17,18]. These processes are primarily regulated by Rho family GTPases, including Rac1 and RhoA, which spatially coordinate actin assembly and contractile forces [8,19].

Importantly, the same molecular regulators govern cell migration, where Rac1 promotes protrusion at the leading edge, and RhoA mediates contraction at the trailing edge, establishing cell polarity and directional motility [19]. This shared molecular machinery suggests a functional coupling between phagocytosis and migration through polarity-regulating mechanisms. Indeed, polarity-dependent signaling has been shown to control directional sensing and cytoskeletal organization in migrating immune cells [20]. However, whether phagocytosis itself generates polarity that governs whole-cell migration and spatial prioritization of engulfment remains unclear.

Recent studies have revealed spatial constraints in phagocytosis. For example, macrophages exhibit spatial resolution limits in discriminating closely spaced opsonized targets, suggesting that phagocytic signaling propagates over finite spatial ranges [21]. Furthermore, internalization of partially opsonized or heterogeneous targets has demonstrated that mechanical factors, including membrane tension, cytoskeletal forces, and target geometry, influence engulfment behavior [22,23]. In addition, recent research on complement receptor (CR) 3-mediated phagocytosis suggests that macrophages localize RhoA to phagocytic cups during phagocytosis, thereby remodeling the actin cytoskeleton [24]. These findings indicate that phagocytosis is not governed solely by local receptor–ligand interactions but also involves global and complex mechanical coordination at the cellular scale.

Our previous microcapillary manipulation experiments demonstrated that macrophages engulf ligand-deficient particles when mechanically coupled to opsonized targets, revealing that mechanical progression of membrane extension can drive internalization beyond ligand-mediated recognition [25,26]. These findings suggested the presence of cell-scale coordination mechanisms that integrate mechanical and biochemical signals during phagocytosis. Furthermore, microneedle-based phagocytosis experiments have shown that membrane extension toward opsonized microneedles is accompanied by directional cell migration, indicating functional coupling between phagocytosis and motility.

Because microneedles provide effectively unlimited antigen geometry and allow precise spatial and temporal control of stimulation, microneedle manipulation offers a powerful platform for investigating spatiotemporal coordination between membrane extension, cell migration, and polarity formation [27]. In particular, microneedles can elicit long-range membrane extension and backtracking dynamics that are difficult to induce and quantify with conventional spherical targets. Such experimental platforms are valuable for understanding immune cells as adaptive biological micromachines that dynamically integrate sensing, force generation, and target processing.

In this study, we investigated whether phagocytic membrane extension generates transient, whole-cell polarity that coordinates migration and the spatial prioritization of engulfment. We used opsonized glass microneedles as precisely controlled model antigens to impose spatially defined stimulation and to quantify long-range membrane extension dynamics. By sequentially stimulating macrophages with multiple microneedles and analyzing engulfment behavior under uniform microbead stimulation, we quantified the temporal coupling between membrane extension and directional migration and mapped how polarity biases the initiation of competing engulfment events. Our results demonstrate that phagocytic membrane extension establishes transient polarity that synchronizes cell migration and spatially gates engulfment, revealing fundamental operational principles of macrophages as adaptive living micromachines.

## 2. Materials and Methods

### 2.1. Cells

We used a mouse-derived macrophage-like cell line (J774.2; Sigma-Aldrich, St. Louis, MO, USA) as the macrophage model. The cells were grown at 37 °C under 5% CO_2_ in Dulbecco’s modified Eagle’s medium (DMEM: Gibco Thermo Fisher Scientific, Waltham, MA, USA) supplemented with 10% heat-inactivated fetal bovine serum (FBS: Gibco Thermo Fisher Scientific, Waltham, MA, USA) and 100 U/mL penicillin–streptomycin (Gibco Thermo Fisher Scientific, Waltham, MA, USA). Given the changes in characteristics with increasing cell passages, the passages were carried up to the fifth generation.

### 2.2. Preparation of IgG-Coated Antigen Samples

We used the same IgG decoration procedure on glass microneedles and polystyrene microbeads as described in our previous reports [25,26,27,28]. Glass microneedles were fabricated from 1 mm glass rods (G-1000; Narishige, Tokyo, Japan) using a micropipette puller (P-97; Sutter Instrument, Novato, CA, USA) and a microforge (MF-2; Narishige, Tokyo, Japan). The fabricated microneedles were incubated in 10 mg/mL bovine serum albumin (BSA; Sigma Aldrich, St. Louis, MO, USA) in phosphate-buffered saline (PBS; Takara Bio, Shiga, Japan) overnight at room temperature, after being washed with PBS three times. After the overnight incubation, the needles were rewashed with PBS three times and then incubated in 0.11 mg/mL anti-albumin IgG (A10-127A; Bethyl Laboratories, Montgomery, TX, USA) in PBS for one hour at room temperature. Finally, the needles were rewashed with PBS once and stored in sufficient PBS at 4 °C. The IgG opsonization onto polystyrene microbeads was performed using the same procedure; 2 × 10^6^ microbeads with a diameter of 10 μm (17136-5; Polysciences, Warrington, PA, USA) were incubated in 10 mg/mL BSA in PBS overnight and in 0.11 mg/mL IgG in PBS for one hour, and stored in sufficient PBS at 4 °C. Before and after each incubation, the microbeads were washed with PBS three times, and incubations were performed at room temperature. We confirmed the IgG opsonization onto the prepared microneedles and microbeads using a fluorescent secondary goat anti-rabbit antibody (Alexa Fluor 488; Abcam, Cambridge, UK).

### 2.3. Observation of IgG-Coated Microneedle Phagocytosis

We fed macrophages one to three IgG-coated microneedles and observed membrane extension and cell migration during phagocytosis. First, 7.5 × 10^4^ macrophages (J774.2) were seeded in a 60 mm cultivation dish (TPP, Trasadingen, Switzerland) with 5 mL DMEM and incubated at 37 °C under 5% CO_2_ for 3 h. Then, the dish was placed on the stage of an inverted microscope (IX70, 40× objective lens; Olympus, Tokyo, Japan), where the temperature was maintained at 37 °C, after the DMEM was replaced with CO_2_ independent medium (Gibco, Thermo Fisher Scientific, Waltham, MA, USA). Up to three opsonized microneedles were placed on three motorized manipulators (MP225A; Sutter Instrument, Novato, CA, USA) and moved to contact macrophages. Phagocytosis and cell migration were recorded using time-lapse imaging at 5 s intervals with a digital camera (1500M-GE; Thorlabs, Newton, NJ, USA).

### 2.4. Observation of IgG-Coated Polystyrene Microbead Phagocytosis

We observed the phagocytic response of macrophages when they were stimulated by multiple IgG-opsonized targets uniformly and simultaneously, using 10 μm IgG-coated polystyrene microbead. First, 7.5 × 10^4^ macrophages were seeded in a 60 mm cultivation dish with 5 mL DMEM and incubated at 37 °C under 5% CO_2_ for 3 h, and the DMEM was replaced with CO_2_-independent medium. Then, 7.5 × 10^5^ IgG-coated microbeads were gently dispersed in the dish, and the dish was placed on the stage of the temperature-controlled microscope. Next, eight IgG-coated microbeads were quickly captured and contacted onto a macrophage with the microcapillary manipulation assay, consisting of a glass microcapillary tube fabricated from 1 mm glass tubes (GD-1; Narishige, Tokyo, Japan) and a pneumatic injector (Celltram 4r Air; Eppendorf, Hamburg, Germany). When the contacted beads detached from the cell surface, we recontacted them with the same cell. The phagocytic response was recorded using time-lapse imaging at 5 s intervals with a digital camera.

### 2.5. Image Analysis

In IgG-coated microneedle phagocytosis, the time course of the progression of the cell membrane and cell movement during phagocytosis was acquired by measuring the extension length of the cell membrane on the microneedles and the displacement of the cell position, defined as the widest part of the cell body, respectively. Cell membrane detection from the 5 s interval time-lapse micrographs was performed using edge detection in ImageJ ver. 1.54p (National Institutes of Health, Bethesda, MD, USA), which we also used in our previous research [26,27]. In short, cell membrane variations were extracted by subtracting adjacent time-lapse micrographs, noise was removed using a Gaussian filter, and then cell edges were detected using a Sobel filter. The same positional criteria were applied consistently across all samples. In IgG-coated bead phagocytosis, the phagocytic response against each contacted bead was evaluated by the required time from the contact to the initiation of phagocytic cup formation (attached phase) and from initiation to closure of the phagocytic cup (engulfing phase).

## 3. Results and Discussion

### 3.1. Synchronous Coupling of Phagocytosis and Cell Migration During Single Opsonized Microneedle Stimulation

**Figure 1:**
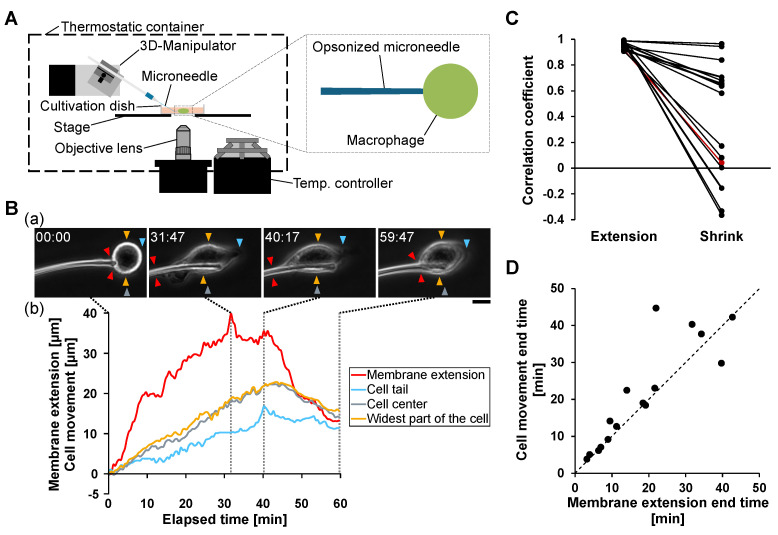
Synchronous coupling of phagocytic membrane extension and directional cell migration during single opsonized microneedle stimulation. (**A**) Schematic illustration of the microneedle manipulation system. The left panel shows the overall experimental setup, comprising a thermostatic chamber, a three-dimensional (3D) micromanipulator, a temperature controller, and an inverted microscopy system. The right panel illustrates the local interaction between an IgG-opsonized glass microneedle and a macrophage inside the cultivation dish. (**B**) Representative example of synchronous membrane extension and cell migration during phagocytosis. (**a**) Time-lapse phase-contrast images showing progressive membrane extension along the microneedle. Red arrowheads indicate the tip of membrane extension, yellow arrowheads indicate the widest part of the cell body, cyan arrowheads indicate the trailing edge, and gray arrowheads indicate the estimated cell center position. Scale bar, 10 μm. (**b**) Time-course analysis of membrane extension length (red), cell tail displacement (cyan), cell center displacement (gray), and widest cell-body position (yellow). (**C**) Correlation analysis between membrane extension and directional cell movement. Correlation coefficients were calculated separately during membrane extension (Extension) and membrane retraction (Shrink) phases for individual cells. The representative example shown in panel B is highlighted with red lines and symbols. (**D**) Temporal relationship between termination of membrane extension and termination of directional cell movement. Each point represents a single cell.

To establish a quantitative platform for analyzing the relationship between phagocytosis and cell migration, we first examined macrophage responses to a single IgG-opsonized glass microneedle (Figure 1A).

Unlike conventional particulate antigens such as opsonized beads or bacteria, opsonized microneedles induce extensive membrane extension along the needle axis, allowing clear observation of coordinated membrane deformation and whole-cell movement. Upon contact with the microneedle, macrophages extended their membranes along the needle surface, marking the onset of phagocytosis (Figure 1B(a)). Concurrently, directional cell migration toward the microneedle was observed.

Quantitative time-course analysis revealed that membrane extension was accompanied by forward displacement of the cell center and trailing edge toward the needle (Figure 1B(b)). During the membrane extension phase, both the cell center and trailing edge moved in the same direction as the extending membrane. In contrast, when membrane retraction began, the direction of cell movement reversed, and the trailing edge shifted away from the microneedle.

To quantitatively evaluate the coupling between membrane extension and cell movement, correlation coefficients were calculated separately during the membrane extension and retraction phases (Figure 1C). Strong positive correlation was observed during membrane extension, indicating tightly coordinated membrane protrusion and cell movement. In contrast, correlation weakened significantly during membrane retraction, indicating decoupling between membrane contraction and directional migration.

We further analyzed the temporal relationship between membrane extension termination and directional cell movement termination. As shown in Figure 1D, the timing of cell movement termination closely followed the timing of maximum membrane extension in 15 of 16 analyzed cells. This relationship indicates that directional cell migration persists while membrane extension progresses and ceases shortly after membrane extension reaches its maximum.

These results demonstrate that phagocytic membrane extension and directional cell migration are coupled in a synchronous manner during antigen engulfment. The initiation, progression, and termination of phagocytosis are closely coordinated with cell motility, suggesting that antigen-induced membrane extension establishes transient motility polarity that governs whole-cell movement.

### 3.2. Coupling of Phagocytosis and Migration Generates Polarity in Response to Sequential Microneedles

**Figure 2:**
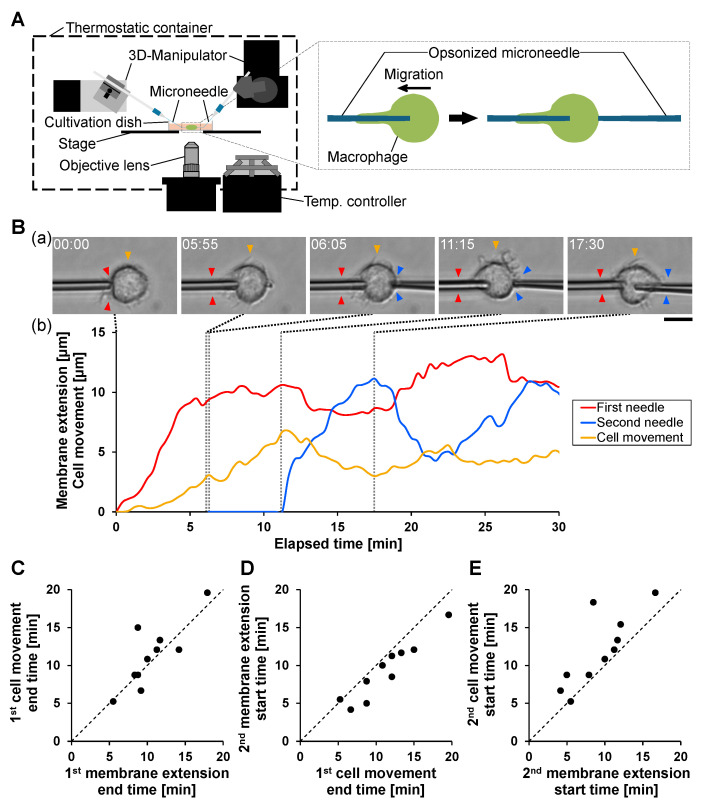
Coupling of phagocytosis and migration generates polarity in response to sequential microneedle stimulation. (**A**) Schematic illustration of sequential stimulation using two opsonized microneedles applied to opposite sides of a macrophage. After phagocytosis was initiated by the first IgG-opsonized microneedle, a second microneedle was attached to the spatially opposite side of the macrophage. (**B**) Representative example of sequential phagocytosis and migration. (**a**) Time-lapse bright-field images showing membrane extension and cell movement. Red and blue arrowheads indicate the tip of membrane extension along the first and second microneedles, respectively. Yellow arrowheads indicate the estimated cell center position based on the widest cell-body location. Scale bar, 10μm. (**b**) Time-course analysis of membrane extension and cell movement. The red and blue lines indicate membrane extensions along the first and second microneedles, respectively. The yellow line indicates the displacement of the cell center. The first and second microneedle contacts occurred at 0 min and 6.3 min, respectively. (**C**) Temporal relationship between the termination of membrane extension along the first needle and the termination of cell movement toward the first needle. Each point represents a single cell (*n* = 10). (**D**) Temporal relationship between termination of cell movement toward the first needle and initiation of membrane extension toward the second needle. (**E**) Temporal relationship between initiation of membrane extension toward the second needle and initiation of directional cell movement toward the second needle.

To investigate how phagocytosis and migration are coordinated under sequential antigen stimulation, we applied a second opsonized microneedle after phagocytosis had been initiated toward the first microneedle (Figure 2A).

A representative example is shown in Figure 2B. Following contact with the first microneedle at 0 min, macrophages extended the membrane along the microneedle and migrated toward it, consistent with the synchronous coupling observed in single-needle stimulation (Figure 1). When the second microneedle contacted the opposite side of the cell at 6.3 min, movement toward the first microneedle persisted. After membrane extension along the first microneedle reached saturation, the migration direction reversed and movement toward the second microneedle began; simultaneously, membrane extension along the second microneedle was initiated. In this example, no prominent retraction of the membrane extension formed along the first microneedle was observed. When membrane extension along the second microneedle reached saturation and transitioned to retraction, the polarity of cell movement reversed again, and the cell resumed movement toward the first microneedle, accompanied by renewed membrane extension along the first microneedle.

To relate the representative behavior to population-level timing, we quantified the temporal relationships among (i) the cessation of membrane extension toward the first microneedle, (ii) the cessation of cell movement toward the first microneedle, and (iii) the initiation of membrane extension and cell movement toward the second microneedle (Figure 2C–E).

Cell movement toward the first microneedle typically ceased shortly after membrane extension toward the first microneedle stopped (Figure 2C), indicating that the extension–migration coupling is preserved under sequential stimulation.

Membrane extension toward the second microneedle generally began slightly before or around the cessation of movement toward the first microneedle (Figure 2D), suggesting that polarity reorganization begins during the late stage of the primary response.

Directional migration toward the second microneedle consistently followed initiation of membrane extension toward the second microneedle (Figure 2E), indicating that membrane extension provides an early indicator of the subsequent migration direction.

These results show that phagocytic membrane extension and directional cell migration remain tightly coordinated under sequential antigen stimulation and that polarity established during primary phagocytosis is dynamically reorganized upon secondary stimulation. The microneedle manipulation platform therefore enables precise spatiotemporal dissection of polarity formation and reorganization during sequential phagocytic events at single-cell resolution.

### 3.3. Analysis of Spatial Distribution of Polarity of Macrophage Phagocytosis Using Third Microneedle Stimulation

**Figure 3:**
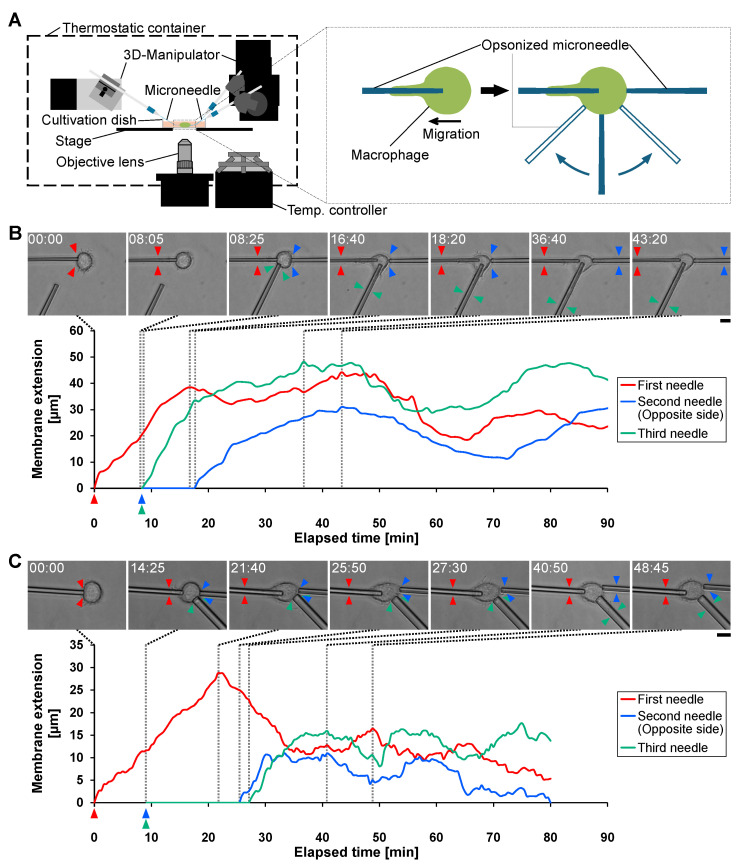
Analysis of spatial distribution of polarity of macrophage phagocytosis using third microneedle stimulation. (**A**) Schematic illustration of the experimental setup and stimulation sequence. The left panel shows the microneedle manipulation system. The right panel illustrates the stimulation procedure. After phagocytosis was initiated by the first opsonized microneedle, the second microneedle was applied to the opposite side of the cell. Simultaneously, the third microneedle was applied either near the first microneedle side or near the second microneedle side to evaluate the spatial distribution of phagocytic responsiveness. (**B**) Representative example where the third microneedle was applied near the first microneedle side. Upper panels show time-lapse bright-field images. Red, blue, and green arrowheads indicate the tip of membrane extension along the first, second, and third microneedle, respectively. Scale bar, 10 μm. The lower graph shows the time-course analysis of membrane extension along each microneedle. Red line indicates extension along the first microneedle, blue line indicates extension along the second microneedle (opposite side), and green line indicates extension along the third microneedle (near the first-needle side). (**C**) Representative example where the third microneedle was applied near the second microneedle side. Upper panels show time-lapse images, and the lower graph shows the corresponding time-course analysis. Scale bar, 10 μm. Red line indicates extension along the first microneedle, blue line indicates extension along the second microneedle, and green line indicates extension along the third microneedle (near the second-needle side).

To assess how phagocytosis-induced polarity is distributed over the cell surface, we introduced a third opsonized microneedle at the time of second-needle stimulation and placed it either near the first-needle side or near the second-needle side (Figure 3A). If phagocytic responsiveness is polarized, a third stimulus delivered near the active extension site should trigger an immediate response, whereas a stimulus delivered near the inhibited side should be delayed.

Figure 3B shows a representative example in which the third microneedle was positioned near the first microneedle. Consistent with the sequential-needle experiments (Figure 2), membrane extension toward the second microneedle was suppressed while phagocytosis toward the first microneedle was ongoing. In contrast, membrane extension toward the third microneedle began promptly after contact, despite ongoing phagocytosis toward the first microneedle.

Figure 3C shows a representative example in which the third microneedle was positioned near the second microneedle. In this configuration, membrane extension toward both the second and third microneedles was suppressed until membrane extension toward the first microneedle approached termination. Membrane extension toward the second and third microneedles then began nearly simultaneously, indicating that the region near the second microneedle was also subject to inhibition.

These results show that polarity established during phagocytosis toward the first microneedle generates spatially heterogeneous phagocytic responsiveness. Regions near the active extension and leading edge retain phagocytic competence and respond rapidly to additional stimulation, whereas regions on the opposite side show delayed responsiveness. This pattern indicates that the phagocytosis-induced polarity produces a spatially extended inhibitory region and thus a gradient of phagocytic competence across the cell surface. Importantly, the microneedle manipulation platform enables direct testing of this spatial regulation by independently controlling antigen stimulation at defined cellular locations.

### 3.4. Spatial Polarity Governs Sequential Phagocytosis Under Uniform Microbead Stimulation

In the previous subsection, microneedle stimulation experiments showed that phagocytosis induces polarity that spatially regulates phagocytic competence, suppressing engulfment on the opposite side while maintaining competence near the active extension site. Here, we asked whether such spatial polarity can also coordinate phagocytosis under uniform antigen exposure, where multiple targets contact the cell surface simultaneously.

**Figure 4:**
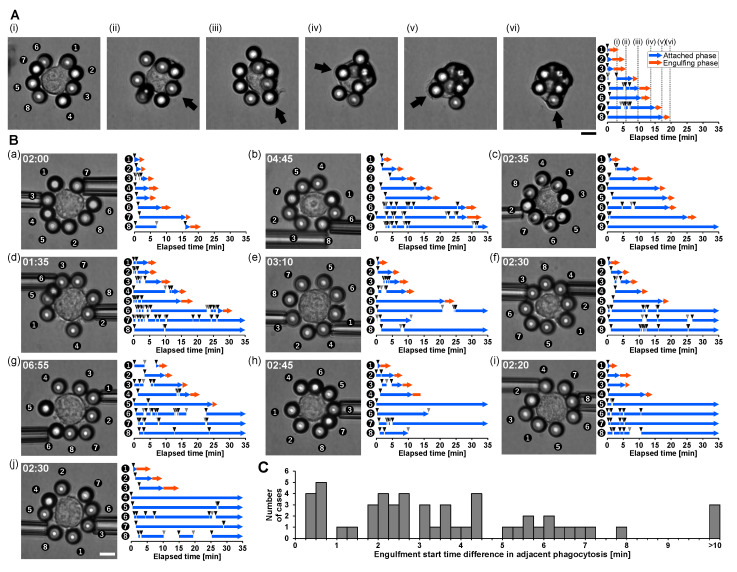
Sequential phagocytosis of uniformly distributed opsonized microbeads reveals polarity-dependent prioritization of engulfment. (**A**) Representative example of phagocytosis of uniformly distributed opsonized microbeads. (**Left panels**) show time-lapse bright-field images of a macrophage surrounded by approximately eight opsonized polystyrene beads (diameter: 10 μm). All beads were positioned around the macrophage using micropipette manipulation and brought into contact with the cell surface within 40 s. Arrows indicate the beads undergoing active engulfment at each time point. Scale bar, 10 μm. The graph on the (**right**) shows the temporal mapping of the attachment and engulfment states for each numbered bead. Blue arrows indicate the attached phase, defined as the period during which a bead remained in contact with the macrophage surface. Red arrows indicate the engulfing phase, defined as the period during which active membrane extension internalizes the bead. Black arrowheads indicate the timing of bead contact, and gray arrowheads indicate the timing of bead detachment from the cell surface. Despite simultaneous exposure and attachment of multiple beads, engulfment occurred sequentially rather than simultaneously. Adjacent beads were engulfed in sequence, whereas some beads remained attached for extended periods without immediate engulfment, demonstrating that engulfment priority is regulated by polarity-dependent spatial competence rather than attachment duration alone. (**B**) (**a**–**j**) Ten additional examples of phagocytosis under uniform microbead stimulation. In each panel, the left image shows the initial bead arrangement, and the right graph shows the temporal mapping of the attachment and engulfment states for each bead. In the fourth phagocytosis in sample (**h**), the cell membrane extended toward the microbeads, but then stopped and backtracked during phagocytosis. Scale bar, 10 μm. (**C**) Distribution of differences in engulfment start times between adjacent phagocytic events. A total of 53 adjacent phagocytic intervals from 11 cells, including the representative example in panel A and 10 additional examples in panel B, were analyzed. No adjacent phagocytic events had an engulfment start-time difference of less than 0.25 min, indicating that simultaneous engulfment was not observed under the present conditions.

Approximately eight opsonized polystyrene beads (diameter: 10 μm) were positioned around a single macrophage using micropipette manipulation and brought into contact within 40 s (Figure 4A). Despite near-simultaneous attachment of multiple beads, engulfment did not occur in parallel. Instead, phagocytosis proceeded sequentially, with only one bead entering the active engulfment phase at a given time.

As shown in Figure 4A, beads located adjacent to each other were engulfed sequentially in order from bead 1 to bead 4. This observation suggests that phagocytic polarity locally promotes engulfment in neighboring regions while suppressing engulfment in spatially distant regions. After several engulfment events, bead positions shifted due to membrane deformation and cell movement, reducing spatial order. However, the one-at-a-time engulfment pattern was consistently preserved.

Engulfment priority was not determined solely by attachment duration. For example, bead 8 remained attached for an extended period without immediate engulfment, indicating that target processing is gated by polarity-dependent spatial competence rather than by the time of contact alone.

These behaviors were also observed in 10 additional samples (Figure 4B). To quantitatively assess the sequentiality of phagocytosis, we analyzed differences in the timing of engulfment initiation between adjacent phagocytic events. Figure 4C shows the distribution of engulfment start time differences for 53 adjacent phagocytic events from 11 cells. Notably, no samples showed an engulfment start time difference of less than 0.25 min, indicating that simultaneous engulfment was not observed under the present conditions. The mean engulfment start time difference was 3.7 min, with a standard deviation of 2.8 min, and the median was 3.1 min. These results quantitatively support the conclusion that macrophages exhibit sequential rather than simultaneous phagocytic responses to uniformly distributed multiple microbeads.

These results demonstrate that macrophages dynamically coordinate phagocytosis through polarity-dependent spatial gating mechanisms. Even under uniform antigen exposure conditions, phagocytosis proceeds sequentially rather than simultaneously, indicating that phagocytic polarity governs both spatial and temporal prioritization of engulfment.

Together with the microneedle stimulation experiments, these findings provide direct experimental evidence that phagocytosis-induced polarity establishes spatially heterogeneous phagocytic competence and regulates sequential processing of multiple targets.

Notably, the microneedle manipulation platform enabled identification of this polarity-dependent regulation, demonstrating its utility for dissecting fundamental mechanisms underlying spatial and temporal coordination of phagocytosis.

### 3.5. Mechanistic Interpretation and Implications

The present microneedle manipulation experiments revealed that phagocytic membrane extension establishes a transient, whole-cell polarity axis that synchronizes directional cell migration and governs the spatial prioritization of engulfment. By enabling long-range membrane extension and subsequent backtracking dynamics, the microneedle system provides a physical basis for how macrophages coordinate phagocytosis across the cell surface.

This polarity-dependent mechanism of spatial regulation is consistent with our earlier observations using an on-chip single-cell cultivation system combined with optical manipulation, in which delayed secondary antigen stimulation did not initiate phagocytosis until completion of the first engulfment process [29]. That study demonstrated that macrophages can integrate sequential antigen stimulation over time and selectively prioritize ongoing phagocytic events, suggesting the existence of global regulatory mechanisms controlling engulfment. However, the physical basis for this prioritization remained unclear. Furthermore, a recent preprint study by Paulson et al. showed an interesting tendency termed ‘phagocytic priming’ that microglia that had phagocytosed IgG-opsonized particles exhibited more unidirectional migration compared to intact microglia [30]. However, they did not focus on the relationship between membrane dynamics and cell migration during phagocytic process, and it remained unclear how a phagocytic event in a single macrophage affects its subsequent phagocytic responses to target stimuli on other sites of the cell surface.

The microneedle-based analysis provides a direct mechanistic explanation for these earlier observations. By enabling membrane extension over distances exceeding the cell radius and revealing subsequent membrane backtracking dynamics, the microneedle system allowed direct observation of how phagocytic membrane extension establishes a polarity field across the cell surface. This polarity promotes engulfment near the active extension site while suppressing engulfment at distant regions. Such polarity-dependent spatial gating explains the delayed initiation of secondary phagocytosis observed in earlier on-chip experiments and supports the conclusion that phagocytosis is regulated as a globally coordinated cellular process rather than as independent local receptor-triggered events. Although microneedles have a geometric shape different from that of typical in vivo targets such as bacteria and particulate matter, spatial gating of phagocytosis similar to that observed in this study may also occur when macrophages engage targets that are too large to be fully engulfed, and membrane extension is prolonged toward the target.

These findings also extend our recent study, which demonstrated spatial-resolution limits in macrophage phagocytosis using micromanipulation techniques [25]. Together, these results indicate that macrophages dynamically regulate phagocytic competence through coordinated allocation of membrane extension capacity and polarity-dependent spatial prioritization. The ability of microneedles to induce prolonged membrane extension and backtracking—phenomena not typically observed with conventional spherical antigen models [26,28]—was essential for revealing this coordinated, polarity-driven regulation.

Mechanistically, the observed polarity-dependent coordination is consistent with established roles of Rho family GTPases in regulating cytoskeletal organization during phagocytosis and migration. Rac1 promotes actin polymerization and membrane protrusion at the leading edge, whereas RhoA mediates contractile forces associated with rear retraction and polarity stabilization [31,32,33]. These molecular systems establish front–rear polarity that governs directional movement and membrane remodeling [8]. Furthermore, mechanical studies have demonstrated that phagocytosis involves coordinated force generation and a spatially organized cytoskeletal machinery that resembles micromachine-like operation [18]. The strong coupling observed in this study between membrane extension, migration, and engulfment prioritization is consistent with such polarity-regulated cytoskeletal coordination.

Furthermore, it has been suggested that the localization of Rac1/RhoA may be switched by the mechanosensing mechanism of the cells. For example, Masters et al. showed that increased membrane tension in macrophages due to osmotic stress led to the cessation of pseudopod extension accompanied by the loss of Rac1 activity in frustrated phagocytosis, but that Rac1 activity at the extension edge was restored and the extension resumed by reducing membrane tension with isotonic solution [34]. Another study showed that in macrophages that had phagocytosed IgG-opsonized particles and formed actin-rich phagosomes, podosomes on the cell surface, involved in cellular mechanosensing, were temporarily disrupted [35]. Taken together, these studies suggest that phagocytosis and Rac1/RhoA-associated polarity may influence each other through changes in membrane tension and in the organization of the cell surface during target internalization.

This interpretation is further supported by our recent microneedle backtracking study, in which we showed that the single-target engulfment ceiling, defined as the maximum membrane extension at the onset of backtracking, remained quantitatively invariant regardless of the intracellular phagocytic load imposed by previously internalized IgG-opsonized beads [36]. That result indicates that local membrane extension toward a single target is regulated independently of total intracellular cargo burden. In contrast, the present study demonstrates that the spatial initiation of subsequent phagocytic events is strongly influenced by the polarity established during ongoing phagocytosis. Together, these findings suggest that macrophage phagocytosis is regulated by at least two coupled but distinct layers: a load-independent local ceiling for single-target membrane extension, and a polarity-dependent global coordination mechanism that governs the spatial prioritization of multiple targets.

Although the present study did not directly measure the intracellular localization or activity of Rac1, RhoA, or related signaling molecules, the observed spatiotemporal coordination suggests that polarity-dependent cytoskeletal regulation underlies the spatial control of phagocytosis. Future studies combining microneedle manipulation with fluorescence imaging of these regulators will be important for testing this hypothesis and elucidating the signaling mechanisms underlying polarity formation and spatial prioritization.

Finally, the microneedle manipulation platform developed in this study provides a unique experimental approach for dissecting the operational principles of macrophages as adaptive living micromachines. In this context, a “micromachine-like” response refers to the integration of localized mechanical input (spatially defined antigen contact), an internal state variable (transient polarity across the cell), and coordinated outputs (directed migration and prioritized engulfment). Unlike conventional particulate antigen models, microneedles allow controlled spatial stimulation and prolonged membrane extension, enabling direct observation of how membrane extension capacity and engulfment priority are dynamically regulated across the cell surface.

Taken together, these results establish a unified mechanistic framework in which phagocytic membrane extension generates transient polarity that governs directional migration and the spatial prioritization of engulfment. This polarity-driven regulation provides a physical basis for spatial discrimination and sequential antigen processing in macrophages and highlights the importance of micromanipulation-based approaches for uncovering fundamental operational principles of living micromachines. Although the present study focuses on macrophage phagocytosis under controlled micromanipulation, the concept of polarity-dependent target prioritization may also provide a useful conceptual framework for future studies of immune-cell behavior in complex tissue or tumor-associated microenvironments, where heterogeneous spatial cues and competing targets coexist [37].

## 4. Conclusions

This study demonstrates that phagocytic membrane extension establishes a transient, whole-cell polarity axis that synchronizes directional migration and governs the spatial prioritization of engulfment in macrophages. Using a microneedle manipulation platform together with uniform microbead stimulation, we showed that ongoing phagocytosis suppresses competing engulfment at distant sites while permitting sequential engulfment in spatially proximal regions, resulting in ordered target processing even under uniform antigen exposure. These findings support the view that macrophage phagocytosis is regulated by polarity-dependent global coordination rather than by independent, purely local receptor-triggered events. The micromanipulation approach developed here provides an experimental framework for dissecting the spatiotemporal regulation of phagocytosis and for understanding macrophages as adaptive living micromachines that integrate mechanical inputs, transient polarity formation, and sequential target processing.

## Data Availability

The original contributions presented in this study are included in the article material. Further inquiries can be directed to the corresponding author.
